# The association between self-reported psychosomatic complaints and bullying victimisation and disability among adolescents in Finland and Sweden

**DOI:** 10.1177/14034948221089769

**Published:** 2022-05-01

**Authors:** Ylva Bjereld, Lilly Augustine, Russell Turner, Petra Löfstedt, Kwok Ng

**Affiliations:** 1 Department of Behavioural Sciences and Learning, Linköping University, Linköping, Sweden; 2 CHILD, Department of Education and Psychology, School of Education and Communication, Jönköping, Sweden; 3 Department of Social Work, University of Gothenburg, Gothenburg, Sweden; 4 Department of Public Health and Community Medicine, University of Gothenburg, Gothenburg, Sweden; 5 Physical Activity for Health Research Centre, Department of Physical Education and Sport Sciences, University of Limerick, Ireland; 6 School of Educational Sciences and Psychology, University of Eastern Finland, Finland

**Keywords:** Bullying, disability, HBSC, self-reported psychosomatic complaints, protective factors

## Abstract

**Aim:**

To analyse the associations between bullying victimisation, disability, and self-reported psychosomatic complaints in adolescents, and to investigate the role of support from parents and teachers in such associations.

**Methods:**

The study was based on Finnish and Swedish data from two waves (2013/2014 and 2017/2018) of the Health Behaviour in School-aged Children survey (*n*=16,057). Descriptive statistics were produced for four groups of adolescents: (a) bullied with disabilities; (b) not bullied with disabilities; (c) bullied without disabilities; and (d) not bullied without disabilities (reference group). Two multilevel multinomial logistic regression models were performed for the Finnish and Swedish samples separately. The first model analysed associations between psychosomatic complaints and bullying victimisation, controlling for a range of confounders. The second model analysed associations between psychosomatic complaints and social support from parents and teachers.

**Results:**

Across both countries, bullied adolescents with disabilities were more likely to self-report psychosomatic complaints than the reference group, even after adjusting for other potential confounders. Teacher support was identified as a potential protective factor as the odds ratio for psychosomatic complaints decreased when including teacher support as a factor in the model. The association with parent support showed mixed findings in Finland and Sweden.

**Conclusions:**

**Disability in combination with bullying victimisation generated the highest levels of self-reported psychosomatic complaints compared to adolescents that were not bullied nor had disabilities. High teacher support may be a protective factor against psychosomatic complaints for bullied and/or disabled adolescents.**

## Introduction

Bullying is a harmful behaviour with negative consequences for the victim and is one of the main reasons why adolescents feel unsafe in school [1]. School bullying can be defined as repeated aggression directed at less powerful students [2]. The international classification of functioning, disability, and health (ICF) of the World Health Organization (WHO) describes disability as a bodily impairment or functional limitation, including difficulties an individual may have in executing activities or problems with involvement in daily life situations [3]. Adolescents with disabilities are at higher risk of being bullied then their non-disabled peers [4]. Bullied adolescents, as well as adolescents with disabilities, have a higher risk of self-reported psychosomatic complaints compared with adolescents who are not bullied or who do not have disabilities [5–8]. Self-reported psychosomatic complaints include both somatic and psychological symptoms and are related to stress, in that they are viewed to increase with greater exposure to stressful experiences [9]. Although bullying and disability are associated with psychosomatic complaints, less is known about the psychosomatic complaints of adolescents who are both bullied and have disabilities. Having several psychosomatic complaints has been related to problems such as loneliness [10], poor friendships, negative class climate, school pressure [9], suicidal ideation and behaviour [11].

Although these studies have mainly focused on risk factors and problems, it is just as important to study factors that may protect adolescents from psychosomatic complaints. Previous research has shown that positive relations with parents and teachers and having the opportunity to talk about bullying with an adult can serve as protective factors for adolescents’ mental health [12, 13]. Few studies have demonstrated whether such positive relations with adults protect bullied and/or adolescents with disabilities from psychosomatic complaints to the same extent as other adolescents. Thus, the aim of the current study was to analyse the associations between bullying victimisation, disability, and self-reported psychosomatic complaints in adolescents, and to investigate the role of support from parents and teachers in such associations.

## Methods

The study was based on Finnish and Swedish data from two waves of the Health Behaviour in School-aged Children (HBSC) survey conducted in winter 2013/2014 and 2017/2018. The HBSC is a WHO collaborative study that distributes a cross-sectional survey every 4 years. In both Finland (FIN) and Sweden (SE), the questionnaire was distributed to adolescents in school years 5, 7 and 9 (mean age 11, 13 and 15 years). No formal approval from an ethical review board was required because the participants completed the questionnaire anonymously and voluntarily. Parents/guardians were informed by schools that the study was going to take place and those who did not want their adolescents to participate were asked to inform the school to opt out. Thus, passive consent was given by parents and the adolescents gave their assent prior to answering the questionnaire. More information on the design and method of the HBSC can be found in the international reports [14, 15].

### Sample

Participants were recruited to the study by using a two-step sampling procedure, which is further described in the HBSC study design protocol [14, 15]. In Finland, 191 schools were invited in 2014, with a 69% (*n*=132) school response rate. In 2018, 221 schools were invited, with 80% (*n*=176) school response rate. The response rate in Finnish schools at the individual level was 84% in 2013/2014 and 64% in 2017/2018, and the decline could be attributed to a lack of willingness to participate. A total of 6040 adolescents in 7th and 9th grades completed the survey. Questions on disabilities were not available for 5th graders in Finland and this age group was removed from analyses.

In Sweden, the response rate for schools was 77% in 2013/2014, and 47% in 2017/2018. One explanation to the drop in the response rate between 2013/2014 and 2017/2018 was due to a change in Swedish legislation; it was not possible to remind schools about the survey by directly contacting schools in 2017/2018, which was the case in the previous data collection. The response rate in Swedish schools at the individual level was 90% in 2013/2014 and 89% in 2017/2018. A total of 12,161 adolescents in Sweden answered the questionnaire.

## Measures

### Bullying victimisation and disability

The survey included a commonly used definition of bullying adapted from the revised Olweus bully/victim questionnaire [16], followed by the question: ‘How often have you been bullied at school in the past couple of months?’ Response options were: I have not been bullied at school in the past couple of months/it has happened once or twice/two or three times a month/about once a week/several times a week. Adolescents bullied two or three times a month or more often were coded as ‘bullied’. The measure of bullying victimisation has been validated across multiple cultural contexts [17].

Disability was measured by two items: (a) Do you have a long-term illness, disability, or medical condition (like diabetes, arthritis, allergy, or cerebral palsy) that has been diagnosed by a doctor? (b) Does your long-term illness, disability or medical condition affect your attendance and participation at school? Positive responses to both items were coded as ‘disabilities’. These items have been tested against the child health and illness profile questionnaire among same age adolescents [18].

The bullying victimisation and disability items were combined into one variable: bullying victimisation and disability (BVD) consisting of four categories of adolescents: (a) bullied with disabilities; (b) not bullied with disabilities; (c) bullied without disabilities; and (d) not bullied without disabilities (reference group).

### Psychosomatic complaints

‘Mental health problems’ is a collective term that encompasses both less serious complaints such as feeling low or nervous, to more serious symptoms that are included in psychiatric diagnoses. Being exposed to bullying is associated with mental health problems [19]. In the present study, psychosomatic complaints were used as a measure of mental health problems by asking how often in the past 6 months the adolescent had experienced: headache, abdominal pain, backache, feeling low, irritability or bad mood, feeling nervous, sleeping difficulties, and dizziness. Adolescents who experienced two or more complaints several times a week or daily were coded as having ‘psychosomatic complaints’. The scale has been validated, with the finding that although the included items reflect both psychological and somatic symptoms, the items together measure a unidimensional latent trait of psychosomatic complaints [20].

### Teacher support

Teacher support was measured by three items: (a) I feel that my teachers accept me as I am; (b) I feel that my teachers care about me as a person; and (c) I feel trust in my teachers. Response options were on a 5-point scale ranging from 1 (strongly agree) to 5 (strongly disagree). Together the items had an internal consistency of α=0.87 (Cronbach’s alpha). A sum score was generated from the three items, allowing no missing answers. The teacher support scale has been found to be a valid measurement with high internal reliability [21].

### Communication with parents

Communication with parents was measured by the question: ‘How easy is it for you to talk to your mother/father/stepmother/stepfather about things that really bother you?’ Response options were on a 4-point scale, ranging from 1 (very easy) to 4 (very difficult). Adolescents finding it very easy or easy to talk to at least one parent or step-parent were coded as having ‘good communication’, whereas adolescents answering it was difficult/very difficult to talk to all parents or step-parents were coded as having ‘poor communication’. Adolescents without any parent/step-parent or with no contact with any parent/step-parent or had not answered the question were excluded (FIN *n*=133; SE *n*=298). The measure has been validated in previous research [22].

## Potential confounding covariates

### Bullying perpetration

It is well known that a small number of bullied adolescents are also perpetrators. There are clear distinctions between pure victims and bully victims [23], and adolescents with disabilities are overrepresented in the role of perpetrator [24]. As the focus in this study was on victimisation, the analysis has been adjusted for perpetration. Bullying perpetration was measured with the question: ‘How often have you taken part in bullying another person(s) at school in the past couple of months?’ Adolescents answering that they had bullied others two or three times a month or more often were coded as ‘perpetrator’. The measure of bullying perpetration has been validated across multiple cultural contexts [17].

### Unemployed parent

Adolescents with unemployed parents have a five times higher risk of socioeconomic deprivation than adolescents in families where parents are employed [25]. Children living in socioeconomically deprived families are more likely to be bullied than their not bullied peers [26]. Parental unemployment was thus included as a variable in which adolescents with one or both parents unemployed and looking for a job were coded as ‘parental unemployment’. The measure of parental unemployment has been validated several times, with a high agreement between children and their parents [27].

### Foreign background

Previous research has shown mixed findings on whether foreign background is associated with bullying. Some studies have found foreign background to be associated with bullying victimisation, and victimisation to be more prevalent for visible minority students [28]. Thus foreign background was included as a possible confounding factor in the analysis. Adolescents who were foreign born, and adolescents who were Swedish/Finnish born with two foreign-born parents were coded as ‘adolescents with foreign background’.

### Gender

Adolescents were asked ‘Are you a girl or boy?’.

### Analysis

The HBSC data has a multilevel structure with school classes as sampling units. Two multilevel multinomial models were thus performed to adjust the analysis for the impact of school class (level 2) on BVD group (level 1). In both models, BVD was used as the dependent variable and psychosomatic complaints was included as an independent variable. In model 1, five variables were included as potential confounding factors (bullying perpetration, gender, parental unemployment, foreign background, and school year). Model 2 also included the variables on social support from adults (teacher support and communication with parents). All included variables in the models were categorical except teacher support, which was treated as a continuous variable. Cases with missing data on disability or bullying victimisation were excluded from the analyses (FIN *n*=249, 4.1%; SE *n*=716, 5.9%). Analyses were conducted using IBM SPSS Statistics 25.

## Results

Table I shows that 171 adolescents were categorised as both bullied and having disabilities (FIN 1.4%; SE 0.9%), 890 adolescents were bullied (FIN 7.9%; SE 4.6%), and 1014 adolescents had disabilities but were not bullied (FIN 5.1%; SE 6.4%). Self-reported psychosomatic complaints were most prevalent in the bullied-disabilities group and least prevalent in the reference group (χ2 *P*<0.001). The multilevel model presented in Table II shows that adolescents who were bullied and/or with disabilities were more likely to have psychosomatic complaints than the reference group, even after adjusting for confounders. All included confounder covariates except gender were statistically significantly associated with the BVD group. For most groups, the odds ratios (ORs) for psychosomatic complaints remained at a similar level even after adjusting for the confounders. This could be interpreted as although the confounders were associated with BVD, they did not affect the association between BVD and psychosomatic complaints (unadjusted ORs for psychosomatic complaints are presented in Table III). One exception to this was the bullied-disabilities group in Finland which had 7.47 higher ORs for psychosomatic complaints compared to the reference group. However, after adjusting for confounders, the ORs reduced to under 5.85. The reduced ORs could indicate that confounding factors had a moderating effect on the relation between psychosomatic complaints and the bullied-disabilities group in Finland.

**Table I.** Sample characteristics of the study population presented separately for bullied and not bullied children with and without a disability in percentage, if not stated otherwise.

Bullied-disability		Bullied	Disability	Ref group
Sweden, *n*	107	527	780	10,031
2013/2014	62.6	50.7	58.5	66.6
2017/2018	37.4	49.3	41.5	33.4
School year:				
5	32.7	38.4	23.2	32.2
7	39.3	32.6	31.4	30.8
9	28.0	29.0	45.4	37.1
Boy	41.3	45.1	44.6	49.9
Psychosomatic complaints	81.9	66.0	62.0	33.7
Poor parent communication	23.0	18.5	14.9	8.0
Bullying perpetration	19.6	13.2	2.4	1.2
Unemployed parent	17.6	11.9	5.7	7.2
Foreign background	13.9	13.7	8.0	10.6
Teacher support mean (SD)	7.0 (3.3)	6.5 (3.1)	6.1 (2.8)	5.2 (2.3)
Finland, *n*	64	363	234	3951
2013/2014	56.3	64.7	59.4	63.9
2017/2018	43.8	35.3	40.6	36.1
School year				
5	n/a	n/a	n/a	n/a
7	51.6	60.9	47.4	49.9
9	48.4	39.1	52.6	50.1
Boy	46.9	52.6	38.9	50.5
Psychosomatic complaints	73.4	52.9	59.5	27.0
Poor parent communication	6.8	20.7	21.5	11.8
Bullying perpetration	28.6	13.6	5.6	2.6
Unemployed parent	37.5	30.9	33.3	24.8
Foreign background	12.5	2.5	2.1	1.5
Teacher support mean (SD)	8.5 (3.9)	8.3 (3.2)	8.1 (3.2)	7.1 (2.5)

**Table II.** Model 1: Two-level multinomial regressions of psychosomatic complaints and associated factors, presented separately for bullied and not bullied children with and without disability in odds ratios (95% confidence interval).

	Sweden			Finland		
	Bullied-disability	Disability	Bullied	Bullied-disability	Disability	Bullied
Psychosomatic complaints						
2 or more	8.40 (4.78–14.72)***	3.14 (2.65–3.71)***	3.87 (3.12–4.81)***	5.85 (3.26–10.49)***	3.61 (2.73–4.78)***	3.06 (2.43–3.86)***
0–1	ref	ref	ref	ref	ref	ref
Bully perpetration						
Bully	12.25 (6.27–23.93)***	1.66 (0.81–3.02)	11.15 (7.58–16.39)***	11.37 (5.89–21.95)***	2.02 (1.09–3.73)*	5.07 (3.44–7.47)***
No bully	ref	ref	ref	ref	ref	ref
Unemployed parent						
Yes	2.17 (1.20–3.93)*	0.85 (0.60–1.20)	1.50 (1.08–2.09)*	0.75 (0.44–1.30)	0.72 (0.54–0.97)*	0.86 (0.68–1.10)
No	ref	ref	ref	ref	ref	ref
Foreign background						
Yes	1.40 (0.86–2.29)	0.71 (0.57–0.89)**	1.08 (0.84–1.39)	4.49 (1.83–11.04)**	1.11 (0.43–2.86)	1.08 (0.51–2.30)
No	ref	ref	ref	ref	ref	ref
School grade						
5	2.38 (1.34–4.23)**	0.68 (0.55–0.84)***	1.96 (1.49–2.56)***	n/a	n/a	n/a
7	2.30 (1.34–3.97)**	0.92 (0.76–1.12)	1.56 (1.19–2.04)**	1.21 (0.72–2.03)	0.90 (0.69–1.19)	1.66 (1.31–2.09)***
9	ref	ref	ref	ref	ref	ref
Sex						
Girl	1.19 (0.75–1.88)	0.99 (0.84–1.17)	1.13 (0.92–1.40)	1.30 (0.74–2.28)	1.28 (0.96–1.71)	0.88 (0.69–1.11)
Boy	ref	ref	ref	ref	ref	ref

Reference group: neither bullied nor having a disability.*P<0.05; **P<0.01; ***P<0.001.

**Table III.** Model 2: Two-level multinomial regressions of psychosomatic complaints and social support from parents and teachers, presented separately for bullied and not bullied children with and without disability in odds ratios (95% confidence interval) in STEP 1 and in adjusted odds ratios in STEP 2.

	Sweden			Finland		
	Bullied-disability	Disability	Bullied	Bullied-disability	Disability	Bullied
STEP 1						
Psychosomatic complaints						
2 or more	8.90 (5.26–15.08)***	3.19 (2.72–3.74)***	3.82 (3.13–4.67)***	7.47 (4.26–13.07)***	3.98 (3.03–5.22)***	3.07 (2.47–3.82)***
0–1	ref	ref	ref	ref	ref	ref
Parent communication						
Poor	3.41 (2.3–5.49)***	2.00 (1.62–2.48)***	2.69 (2.12–3.42)***	0.54 (0.20–1.50)	2.04 (1.46–2.85)	1.96 (1.49–2.58)
Good	ref	ref	ref	ref	ref	ref
Low teacher support	1.26 (1.19–1.34)***	1.14 (1.11–1.17)***	1.21 (1.17–1.24)***	1.20 (1.10–1.30)	1.14 (1.09–1.19)	1.17 (1.13–1.22)
STEP 2						
Psychosomatic complaints						
2 or more	5.78 (3.33–10.03)***	2.89 (2.44–3.42)***	3.14 (2.54–3.90)***	6.46 (3.59–11.62)***	3.47 (2.60–4.64)***	2.48 (1.97–3.14)***
0–1	ref	ref	ref	ref	ref	ref
Parent communication						
Poor	2.20 (1.33–3.64)**	1.28 (1.01–1.62)*	1.64 (1.26–2.15)***	0.30 (0.11–0.86)*	1.26 (0.88–1.80)	1.30 (0.97–1.74)
Good	ref	ref	ref	ref	ref	ref
Low teacher support	1.14 (1.06–1.23)***	1.08 (1.05–1.12)***	1.14 (1.10–1.18)***	1.10 (1.00–1.21)	1.06 (1.00–1.11)*	1.12 (1.07–1.17)***

Reference group: neither bullied nor having a disability. *P<0.05; **P<0.01; ***P<0.001.

### Self-reported psychosomatic complaints and social support from teacher and parents

Adolescents who were bullied and/or with disabilities had higher ORs for having low teacher support, compared to the reference group (Table III). The difference was statistically significant for all groups except the bullied-disabled group in the Finnish sample. In Sweden, there was a significant association between poor communication with parents and BVD, with the highest adjusted ORs in the bullied-disabilities group (adjusted OR 5.78). The Finnish result for adolescents who were solely bullied or had disabilities pointed in the same direction as the Swedish, but it was not statistically significant. The bullied-disabilities group in Finland had an opposite result to the Swedish group: Finnish adolescents that were both bullied and had disabilities had lower ORs than the reference group for poor communication with the parents. In step two of the model when the covariates were adjusted for each other, the ORs for psychosomatic complaints decreased. This could be interpreted as teacher support and parent communication had a moderating impact on the association between BVD and psychosomatic complaints in both countries.

## Discussion

Although previous research has established a connection between bullying and psychosomatic complaints [5, 6], as well as between disabilities and psychosomatic complaints [7, 8], there is an empirical knowledge gap concerning bullied adolescents with disabilities and the associations with psychosomatic complaints. The current study addressed this gap by showing that bullied adolescents with disabilities had the highest levels of self-reported psychosomatic complaints compared to peers who were not bullied nor had disabilities, suggesting that a double marginalisation of being both bullied and having disabilities increased the association with psychosomatic complaints.

Explanations for the higher ORs for psychosomatic complaints among bullied and adolescents with disabilities, and especially adolescents who had the combination of both, can be made using the ICF model [3]. In the ICF model, bullying can be viewed as an environmental barrier, in which there is a lack of support from family and negative attitudes of peers that restricts the victimised adolescent’s functioning. Disability, as measured in the current study, included bodily limitations that affected adolescents’ attendance and participation at school. Thus the interaction between the bodily (impairments/health conditions) and environmental (bullying) barriers reduced health and functioning (psychosomatic complaints) more than one barrier alone did.

This study also explored whether protective relations with adults, measured as teacher support and good parental communication, adjusted the ORs for psychosomatic complaints. High teacher support was associated with less bullying and was statistically significant in all groups except for the group of adolescents that were both bullied and had disabilities in Finland. When teacher support was included in the model, the ORs for psychosomatic complaints decreased. Hence being supported by a teacher can be interpreted as a protective factor for self-reported psychosomatic complaints in bullied/and or adolescents with disabilities. When an adolescent is identified as bullied in combination with disability, there is an evident risk that the child suffers from several psychosomatic complaints. Improving protective relations may be an important target for intervention concerning bullied adolescents with disabilities.

In this study, we assumed that good parental communication would have a similar association to teacher support, that is, be associated with less bullying. Although this expectation was true for the Swedish sample, the Finnish result was different. In Finland, BVD was not significantly associated with parental communication in all groups. For the bullied-disabilities group, the association was opposite to expectation, that is, good communication with parents was associated with higher ORs for being bullied. The current study does not provide any answers to why there was an intercountry difference for the association between parental communication and BVD. However, there are two suggestions that might be part of the explanation. The first hypothesis is that the difference is related to the national anti-bullying work. In Finland, 90% of the schools are using the anti-bullying programme KiVa, which has a documented effect on bullying prevalence [29]. In Sweden, schools are obliged to work against all forms of peer harassment (the Swedish Education Act 2010:800). Yet Swedish schools are not specifically advised to use anti-bulling programmes, and schools are thus managing bullying in various ways. Further, one component of the Finnish KiVa programme includes informing parents about bullying and the anti-bullying work, but similar information is not given to the same extent to parents in Sweden, as it is up to individual schools if they inform parents at all. Potentially, the parent component in the KiVa programme encourages Finnish adolescents to talk about bullying with their parents in another way than Swedish adolescents and parents do. The second part of the explanation might be found in the school attendance of adolescents with disabilities. In Sweden, adolescents with disabilities attend general schools, regardless of need, whereas in Finland school and class allocation of adolescents with disabilities is based on classifications of their support needs [30]. As such, there might be some intercountry differences due to school organisational factors.

There are some limitations to the present study. As the data were cross-sectional, no causal link between teacher support/parent communication and psychosomatic complaints can be made from the current analysis. It is possible that adolescents with less severe disabilities also had the strongest relationships with parents and teachers.

In the present study, the disability measure included a few examples to exemplify what was meant by a diagnosis. It is possible that children with a condition that was not exemplified in the measure, such as epilepsy, did not report this as a diagnosis. Further, the measure did not distinguish between various disabilities. Instead, a condition was categorised as disability if it led to reduced participation at school or in daily life. Several disabilities were thus combined in the measure, including conditions of varying severity.

At the time the HBSC questionnaire was administered, there was no item on neutral or non-binary gender response categories which should be considered a limitation. Finally, the response rate for Finnish and Swedish schools was low in 2017/2018. However, as the non-response was random and the sample size large, it is unlikely that the low response rate had a significant impact on the results.

## Conclusions

Adolescents who were bullied and/or with disabilities had higher ORs for having self-reported psychosomatic complaints, compared to their peers who were not bullied and without disabilities. Disability in combination with bullying victimisation generated the highest ORs of psychosomatic complaints. High levels of teacher support were identified as a protective factor for psychosomatic complaints in bullied and/or disabled adolescents.
